# Non-linear registration improves statistical power to detect hippocampal atrophy in aging and dementia

**DOI:** 10.1016/j.nicl.2019.101902

**Published:** 2019-06-18

**Authors:** F. Bartel, M. Visser, M. de Ruiter, J. Belderbos, F. Barkhof, H. Vrenken, J.C. de Munck, M. van Herk, for the Alzheimer's Disease Neuroimaging Initiative

**Affiliations:** a Department of Radiology and Nuclear Medicine, VU University Medical Center, Amsterdam, the Netherlands; b Division of Psychosocial Research and Epidemiology, Netherlands Cancer Institute, Amsterdam, the Netherlands; c Department of Radiotherapy, Netherlands Cancer Institute, Amsterdam, the Netherlands; d UCL institutes of Neurology and healthcare engineering, London, United Kingdom; e Manchester Cancer Research Centre, Division of Cancer Science, School of Medical Sciences, Faculty of Biology, Medicine and Health, University of Manchester, Manchester Academic Health Sciences Centre, Manchester, United Kingdom

**Keywords:** Longitudinal MRI, MCI, AD, Hippocampal atrophy, Non-linear registration, Automatic segmentation

## Abstract

**Objective:**

To compare the performance of different methods for determining hippocampal atrophy rates using longitudinal MRI scans in aging and Alzheimer's disease (AD).

**Background:**

Quantifying hippocampal atrophy caused by neurodegenerative diseases is important to follow the course of the disease. In dementia, the efficacy of new therapies can be partially assessed by measuring their effect on hippocampal atrophy. In radiotherapy, the quantification of radiation-induced hippocampal volume loss is of interest to quantify radiation damage. We evaluated plausibility, reproducibility and sensitivity of eight commonly used methods to determine hippocampal atrophy rates using test-retest scans.

**Materials and methods:**

Manual, FSL-FIRST, FreeSurfer, multi-atlas segmentation (MALF) and non-linear registration methods (Elastix, NiftyReg, ANTs and MIRTK) were used to determine hippocampal atrophy rates on longitudinal T1-weighted MRI from the ADNI database. Appropriate parameters for the non-linear registration methods were determined using a small training dataset (N = 16) in which two-year hippocampal atrophy was measured using test-retest scans of 8 subjects with low and 8 subjects with high atrophy rates. On a larger dataset of 20 controls, 40 mild cognitive impairment (MCI) and 20  AD patients, one-year hippocampal atrophy rates were measured. A repeated measures ANOVA analysis was performed to determine differences between controls, MCI and AD patients. For each method we calculated effect sizes and the required sample sizes to detect one-year volume change between controls and MCI (N_CTRL_MCI_) and between controls and AD (N_CTRL_AD_). Finally, reproducibility of hippocampal atrophy rates was assessed using within-session rescans and expressed as an average distance measure D_Ave_, which expresses the difference in atrophy rate, averaged over all subjects. The same D_Ave_ was used to determine the agreement between different methods.

**Results:**

Except for MALF, all methods detected a significant group difference between CTRL and AD, but none could find a significant difference between the CTRL and MCI. FreeSurfer and MIRTK required the lowest sample sizes (FreeSurfer: N_CTRL_MCI_ = 115, N_CTRL_AD_ = 17 with D_Ave_ = 3.26%; MIRTK: N_CTRL_MCI_ = 97, N_CTRL_AD_ = 11 with D_Ave_ = 3.76%), while ANTs was most reproducible (N_CTRL_MCI_ = 162, N_CTRL_AD_ = 37 with D_Ave_ = 1.06%), followed by Elastix (N_CTRL_MCI_ = 226, N_CTRL_AD_ = 15 with D_Ave_ = 1.78%) and NiftyReg (N_CTRL_MCI_ = 193, N_CTRL_AD_ = 14 with D_Ave_ = 2.11%). Manually measured hippocampal atrophy rates required largest sample sizes to detect volume change and were poorly reproduced (N_CTRL_MCI_ = 452, N_CTRL_AD_ = 87 with D_Ave_ = 12.39%). Atrophy rates of non-linear registration methods also agreed best with each other.

**Discussion and conclusion:**

Non-linear registration methods were most consistent in determining hippocampal atrophy and because of their better reproducibility, methods, such as ANTs, Elastix and NiftyReg, are preferred for determining hippocampal atrophy rates on longitudinal MRI. Since performances of non-linear registration methods are well comparable, the preferred method would mostly depend on computational efficiency.

## Highlights

• Registration-based showed highest sensitivity in terms of predicted sample size.
• Registration-based methods reproduce hippocampal atrophy rates best.
• FSL-FIRST and manual segmentations scored lowest in these aspects.

## 1 Introduction

The hippocampus is a small cortical structure that plays an important role in memory formation. In many neurodegenerative diseases it is impaired due to progressive degeneration and/or death of nerve cells which is reflected in a decrease in hippocampal volume. Hippocampal atrophy has been extensively studied in Alzheimer's disease (AD), where neurodegeneration leads to structural brain changes visible on MRI. Hippocampal atrophy is typically determined by delineating the hippocampus (manually or automatically) on longitudinal MRI scans.

Hippocampal volume and volume change have been studied extensively. For instance, hippocampal atrophy has been reported to be larger in AD compared to mild cognitive impairment (MCI) or healthy controls [Apostolova and Thomson, 2009; Henneman et al., 2009; Likeman et al., 2005; Mouiha and Duchesne, 2011; Sabuncu, 2011; Schuff et al., 2009]. In clinical trials, in which disease-modifying therapies are studied, hippocampal atrophy is an important biomarker to provide evidence of treatment effect and to better understand its underlying mechanism. Completed clinical trials in which hippocampal atrophy is an outcome measure are reviewed in [Cash et al., 2014].

Recently, there has also been an interest in investigating hippocampal damage after radiotherapy. Animal studies have shown that the neural stem cell (NSC) compartment in the dentate gyrus of the hippocampus is vulnerable to radiation toxicity and already small doses can damage the NSC [Ferrer et al., 1993; Madsen et al., 2003; Mizumatsu et al., 2003; Nagai et al., 2000; Raber et al., 2004]. Often, in patients with brain tumours or brain metastases brain radiation therapy is the core treatment [Makale et al., 2016]. Furthermore, in patients with small cell lung cancer (SCLC) prophylactic cranial irradiation (PCI) is used to treat microscopic brain disease that is statistically likely to be present, and to reduce the risk of developing larger metastases [Gondi et al., 2010; Péchoux et al., 2016]. The magnitude of hippocampal volume loss due to PCI or other brain radiation treatment techniques is currently unknown. For this reason, it is important to assess and compare the sensitivity of currently available processing techniques to detect volume differences in hippocampal volume from longitudinal MRI.

The hippocampus has limited contrast on MR images because adjacent structures have similar intensities [Fischl et al., 2002] and therefore manual delineation is labour intensive and difficult even for experienced observers. Furthermore, this lack of contrast also is an important source of intra- and inter-observer variability. Measuring hippocampal atrophy rates on longitudinal MRI scans is even more challenging, because in segmentations performed on multiple time-points the volume errors add up, whereas the volume change is small. This was shown by [Mulder et al., 2014], in which manually measured atrophy rates were not well reproduced using longitudinal data with within-session rescans. To avoid the burden of manual labour and to reduce observer variability, automatic segmentation methods have been proposed, most of them reviewed in [Dill et al., 2015] and [González-Villà et al., 2016]. For instance, FSL-FIRST [Patenaude et al., 2011] and FreeSurfer [Fischl et al., 2002; Reuter et al., 2012] are automatic segmentation methods which are used extensively in the academic community. Both methods were investigated in a longitudinal setting in [Mulder et al., 2014] and both showed similarly poor atrophy reproducibility rates. More recently, multi-atlas registration methods were introduced and showed high overlap with manual segmentations [Wang et al., 2013; Zhu et al., 2017] and outperformed FSL-FIRST or FreeSurfer [Pipitone et al., 2014], but these can require long computation times.

An alternative for dedicated longitudinal segmentation methods and multi-atlas registration techniques is to use general purpose non-linear registration algorithms that map a (manual or automatic) segmentation of a baseline (BL) image to a follow up (FU) image. In the presence of MCI and AD, hippocampal atrophy rates have been measured previously using non-linear registration [Crum et al., 2001; Henneman et al., 2009; van de Pol et al., 2007a]. In these studies, atrophy rates measured on the basis of non-linear registration showed improved reliability compared to manually measured atrophy rates and significantly different hippocampal atrophy rates between healthy controls, MCI and AD were found. Hippocampal atrophy rates have been measured using symmetric non-linear registration [Yushkevich et al., 2010] and [Das et al., 2012], which yielded higher sensitivity than a semi-automatic segmentation method [Yushkevich et al., 2010]. Symmetric non-linear registration methods are not susceptible to directional registration bias and are therefore the preferred registration scheme for robust and sensitive longitudinal analysis. A symmetric registration procedure is for instance also used in FreeSurfer's longitudinal pipeline [Reuter et al., 2010; Reuter et al., 2012; Reuter and Fischl, 2011].

In this study, we compared hippocampal atrophy measurements using eight different methods: manual segmentation, automatic segmentation (FreeSurfer v6.0, FSL-FIRST v5.0.10, multi-atlas segmentation with joint label fusion (MALF, [Wang et al., 2013])), and four symmetric non-linear registration methods (Elastix [Klein et al., 2010; Shamonin et al., 2013], NiftyReg [Modat et al., 2010; Modat et al., 2012], Medical Image Registration ToolKit (MIRTK) [Schuh et al., 2014] and the diffeomorphic registration method from the Advanced Normalization Tools (ANTs, http://stnava.github.io/ANTs/) referred to as Symmetric Normalization (SyN) [Avants et al., 2008]). We chose these registration methods because they are publically available and have been frequently used in the academic community. Furthermore, ANTs and MIRTK scored high in a study in which 14 non-linear registration methods were compared [Klein et al., 2009].

The aim of this study was to determine the accuracy of methods to measure subtle hippocampal volume change on the basis of plausibility (does the method show atrophy where biologically expected?), reproducibility (does the method provide the same atrophy rates for within-session rescans of the same subject?) and sensitivity (how many subjects are required to detect a significant group difference?). Therefore, we measured atrophy rates in different diagnostic groups (controls, MCI and AD) and performed an atrophy rate reproducibility analysis.

## 2 Materials and methods

### 2.1 Datasets and image acquisition

In this study we determined hippocampal volume change on two different datasets from the Alzheimer's Disease Neuroimaging Initiative (ADNI) database. Both datasets are described below. The ADNI was launched in 2003 as a public-private partnership, led by Principal Investigator Michael W. Weiner, MD. The primary goal of ADNI has been to test whether serial magnetic resonance imaging (MRI), positron emission tomography (PET), other biological markers, and clinical and neuropsychological assessment can be combined to measure the progression of mild cognitive impairment (MCI) and early Alzheimer's disease (AD).

#### 2.1.1 Training dataset

Training data consisted of a small subset (N = 16) of the ADNI data base in which MCI subjects were recorded at BL and at FU two years later. The same data set was previously used and described in more detail in [Nho et al., 2013]. These data were used to tune parameter settings for each non-linear registration method.

At each time-point, two sagittal 3D T1 weighted magnetization prepared rapid acquisition gradient echo sequence (MPRAGE) 1.5 T MRI were acquired in a single session, just a few seconds apart from each other. In the remainder of this paper we use the term “A and B scans” for those back-to-back (BTB) scan pairs. As described in [Nho et al., 2013], an extreme-trait design was used, where participants were selected at the extremes of the 2-year longitudinal change distribution of hippocampal volume (eight participants with fast rates of atrophy and eight with slow rates of atrophy). The MRI acquisition is explained in more detail in [Jack et al., 2008]. “Gold standard” manual hippocampus segmentations were not available for this dataset. The magnitude of the rate of hippocampal volume change was not reported, but groups (slow and fast) differed in the rate of change (p < 0.001) determined by the statistical parametric mapping (SPM) software package (http://www.fil.ion.ucl.ac.uk/spm/).

#### 2.1.2 Validation dataset

This dataset is the same dataset as used by [Mulder et al., 2014] and [Bartel et al., 2017]. Eighty subjects were collected from ADNI including 20 healthy controls (CTRL), 40 MCI and 20 AD subjects. MCI patients were selected based on their CSF profile, using an AD-positive cut-off values of tau/Aβ_1–42_ ≥ 0.39 determined by (Shaw et al., 2009). In (Mulder et al., 2014) two subgroups of MCI patients were a priori selected, 20 MCI patients with an AD-positive CSF profile, and 20 MCI patients with an AD-negative profile (tau/Aβ_1–42_ < 0.39). In the present article, for simplicity those subgroups were merged. Subjects in the CTRL group all had a tau/Aβ_1–42_ value of <0.39. In the AD group, all but one subject had a tau/Aβ_1–42_ value of ≥0.39. The subjects included in the original study (Mulder et al., 2014) were not selected to sample the distribution of disease durations in a systematic fashion or in a fashion representative of the full ADNI cohort and therefore, these data do not allow an investigation of the relation between performance of methods and disease duration. More information about the subjects' demographics is presented in (Mulder et al., 2014). BL and FU scans were obtained one year apart and similarly as for the training dataset BTB scans (A and B scans) were acquired with the same imaging sequence within a single session. The MRI acquisition is explained in [Jack et al., 2008].

### 2.2 Hippocampus segmentation

#### 2.2.1 Manual hippocampus segmentation (only validation dataset)

For the ADNI dataset, hippocampi were segmented at the VU University Medical Center (VUmc) Amsterdam) using the outlining protocol from [Jack, 1994], described in [Jack, 1994; Mulder et al., 2014; van de Pol et al., 2007b]. All BL MRI scans were reformatted perpendicularly to the long axis of the hippocampus with a slice thickness of 2 mm, while the in-plane resolution was kept. The M12 scans were then rigidly registered to the BL scans. For hippocampus segmentation on the M12 scans, BL scans and hippocampus segmentations were shown next to the M12 scans. However, to avoid any training effect the observer (N = 1) obtained A and B scans in a random order and did not know the diagnosis. The observer was a well-trained expert of the VUmc and used in-house developed software (Show_Images 3.7.1.0) for hippocampus segmentation.

#### 2.2.2 FSL v.5.0.10 (both datasets)

FSL-FIRST hippocampus segmentation is described in detail in [Patenaude, 2007] and [Patenaude et al., 2011]. It uses shape and appearance models created from a set of manual hippocampus segmentations from the Center for Morphometric Analysis (CMA), Massachusetts General Hospital (MGH) Boston. The manual segmentations were converted to parameterized surface meshes using intensity values around the tissue border and from these a point distribution model was created. For hippocampus segmentation the MRI is registered to MNI152 standard space using a two-stage affine registration. Then, FSL-FIRST searches through linear combinations of shape variation modes to find the most probable shape by using the intensity values of the MRI. The hippocampal mesh is then converted to a voxel-wise segmentation using FAST [Zhang et al., 2001]. For both datasets, we used the run_first_all command without pre-processing the images.

#### 2.2.3 FreeSurfer v.6.0 (only validation dataset)

FreeSurfer subcortical segmentation is described in detail in [Fischl et al., 2002]. FreeSurfer converts MRI scans to their own conformed 1mm^3^ 256^3^ space, performs a bias-field correction, intensity normalization and skull-stripping for an atlas registration. Using prior intensity and tissue class information, voxels are assigned to subcortical structures. We used FreeSurfer's longitudinal stream to determine hippocampal volumes [Reuter et al., 2010; Reuter et al., 2012; Reuter and Fischl, 2011], which includes an unbiased registration procedure and a label fusion technique. FreeSurfer's longitudinal pipeline was introduced in 2007 (FreeSurfer v4.0.0) and since then FreeSurfer has undergone several improvements. We used FreeSurfer v.6.0 released in 2017. In this study, FreeSurfer's default parameters have not been changed and therefore FreeSurfer was only applied on the validation dataset.

#### 2.2.4 Multi-atlas label fusion segmentation (both datasets)

We used the multi-atlas joint label fusion (MALF) segmentation described in [Wang et al., 2013], implemented in the ANTs software [Avants et al., 2014]. Briefly, using non-linear registration a set of segmented atlases is deformed to a target image and all transformed atlases are combined to one label using a joint label fusion technique [Wang et al., 2013]. To speed up registration time we used a registration scheme provided by the ANTs software (antsRegistrationSyNQuick.sh [Avants et al., 2012; Tustison and Avants, 2013]), which uses a mutual information metric. Twenty atlases were used as input for MALF (9CTRL, 8MCI, 3AD). The segmentation files and MRI were obtained from the Harmonized Protocol for Hippocampal Segmentation (HarP) project's website (http://www.hippocampal-protocol.net/). HarP is a standardized hippocampus outlining protocol in which hippocampal boundary definitions from different outlining protocols were merged [Boccardi et al., 2011; Boccardi et al., 2015; Frisoni et al., 2015].

#### 2.2.5 Non-linear registration methods (both datasets)

As input to non-linear registration, a rigid 6 degree of freedom (DOF) registration was provided that maps the FU scan to the corresponding BL scan. These registrations were all performed using FLIRT from the FSL toolbox [Jenkinson et al., 2002; Jenkinson and Smith, 2001]. Furthermore, brain masks were used for source and target image that were obtained by applying FSL's brain extraction tool (BET) on all subjects' MRI scans. Four symmetric non-linear registration methods were used:

1. Elastix v4.801 [Klein et al., 2010; Shamonin et al., 2013]
2. NiftyReg v1.4.0 [Modat et al., 2010; Modat et al., 2012]
3. Medical image registration toolkit (MIRTK, compiled from the git development tree https://github.com/schuhschuh/MIRTK/tree/develop rev daf2b89, built on Dec 192,017) [Schuh et al., 2014]
4. Diffeomorphic registration method from ANTs v2.2.0, referred to as symmetric normalization (SyN) [Avants et al., 2008].

ANTs-SyN is a symmetric diffeomorphic mapping which guarantees topology preservation. Elastix, NiftyReg and MIRTK are based on free-form deformations (FFD) given by the parameters of a cubic B-spline function [Rueckert et al., 1999]. MIRTK's and NiftyReg's symmetric registration schemes are similar in approach using a symmetric energy formulation to ensure that the transformation is a diffeomorphism [Modat et al., 2012; Schuh et al., 2014]. The symmetric registration approach for Elastix is described in [Metz et al., 2011]. In this approach FU scans are resampled to BL scans using rigid registration and spline interpolation. Then, images were transformed to an average space and the inverse transformation is approximated as described in [Metz et al., 2011]. This is different to diffeomorphic mapping, in which the inverse is guaranteed to exist. Commands and parameters for each registration method are presented in the supplementary files.

#### 2.2.6 Surface reconstruction and mesh deformation

For the validation data set, hippocampi were manually segmented in cropped MR images, reformatted along the long hippocampal axis. We performed our analysis using the native MR images and therefore hippocampus segmentation needed to be mapped back from “segmentation space” to native MRI scan space. To avoid interpolation errors in this procedure, we converted manual segmentation to meshes using the marching cube algorithm [Lorensen and Cline, 1987] and applied linear transformation parameters directly on the meshes. FSL-FIRST, FreeSurfer and MALF were performed on the native MRIs, but for consistency we also converted the hippocampal segmentations obtained from these methods to meshes and computed hippocampal volumes from these meshes.

In the validation set, nonlinearly transformed manual hippocampus meshes were used to determine longitudinal volume changes and thus atrophy rates. For the training dataset manual hippocampus segmentations were not available, therefore FSL-FIRST hippocampus segmentations from the BL scans were used to provide the baseline mesh. The sensitivity of the result to segmentation errors will be estimated below.

### 2.3 Analysis

Hippocampal volumes were obtained by summing signed tetrahedrons created for each triangle in the mesh as described in Cha Zhang and Tsuhan Chen, 2001. Hippocampal atrophy rates were expressed as longitudinal percentage volume change (PVC) defined by:

(1) $$\mathrm{PVC}\left(V_{\mathrm{BL}},V_{\mathrm{FU}}\right)=\frac{V_{\mathrm{FU}}-V_{\mathrm{BL}}}{V_{\mathrm{BL}}}*100$$

with *V*_*BL*_ and *V*_*FU*_ being the volume of the structure from the BL and FU scans, respectively. We calculated PVC for the left and right hippocampus separately, but we averaged them to obtain the subject hippocampal atrophy rates for our analysis. The statistical analysis was performed with IBM SPSS Statistics for Windows v. 22 Armonk, NY: IBM Corp. We analysed atrophy differences between diagnostic groups and atrophy reproducibility using A and B scans. For our statistical analysis we reported and excluded all subjects in which hippocampal PVC was larger than +25% or smaller than −25%, because hippocampal atrophy ±25% is an indicator for poor segmentation or registration and would reduce reliability of the analysis.

#### 2.3.1 Training dataset

For the training dataset “gold standard” manual segmentations were not available. Instead, FSL-FIRST BL segmentations were mapped to FU scans with the registration methods. We report two-year PVC in boxplots to graphically illustrate PVC differences between methods and diagnostic groups. In correspondence with the developers of each registration method we tested different registration parameters until we obtained approximately similar PVC in groups for each method, knowing that the ‘slow’ group should have less atrophy than the ‘fast’ group [Nho et al., 2013]. Using PVC measurements from the A and B scans, with repeated measures ANOVA we determined whether there was a significant difference between the ‘slow’ and the ‘fast’ group (significance level α = 0.05), which indicated if registration methods detected similar atrophy rates.

To be reproducible, the PVCs measured for the A scans should be approximately the same as for the B scans. To study and quantify reproducibility, for each method we plotted PVC values from the A scans against PVC values from the B scans and used an average distance for quantification:

(2) $$D_{\mathrm{Ave},\mathrm{Scans}}=\sqrt{\frac{1}{n}\sum_{i=1}^{n}{\left({\mathrm{PVC}}_{\mathrm{AScan},i}-{\mathrm{PVC}}_{\mathrm{BScan},i}\right)}^{2}}$$

in which *n* is the number of measured PVC values. These “distances” can be interpreted as the root mean squared difference between the A and B scans.

Furthermore, we performed some consistency checks. A commonly used method to determine volume change of a non-linear deformation field is integration of the local Jacobian determinants of the field. For comparison, we determined volume changes using Jacobian integration, which should give very similar results to our mesh-based approach. Finally, we investigated if PVC values depend on the specific choice of the BL segmentation by replacing the FSL-FIRST by the MALF BL segmentation.

#### 2.3.2 Validation dataset

For the validation dataset we used the registration parameter settings determined with the training set and performed a similar analysis as for the training dataset. We report one-year PVC in mean, standard deviations (SD) and boxplots for all methods and diagnostic groups. Differences between CTRL, MCI and AD were assessed with repeated measures ANOVA using Tukey's honestly significant difference (HSD) post hoc test. Furthermore, we performed a power analysis and estimated the sample size needed to detect a reduction in atrophy between patient groups using:

(3) $$N=\frac{2{\left(z_{\alpha /2}+z_{1-\beta }\right)}^{2}}{{\left(\mathrm{Effect Size}\right)}^{2}}=\frac{2{\left(z_{\alpha /2}+z_{1-\beta }\right)}^{2}}{{\left(\frac{\mu_{2}-\mu_{1}}{\sigma_{\mathrm{pooled}}}\right)}^{2}}$$

where μ_i_ are the means of the two groups that are compared (CTRL, MCI or AD). Statistical power (1-β) was set to 80%, significance level α = 0.05 using a two-sided alternative hypothesis. Z_t_ is the t-th quantile of the normal distribution, i.e. z_α/2_ = 1.96 and z_1-β_ = 0.8416. We also presented the atrophy reproducibility measure (D_Ave, Scans_) using A and B scans' determined PVC values and we used the average distance measure to quantify the agreement between different methods to determine atrophy rates:

(4) $$D_{\mathrm{Ave},\mathrm{Method}}=\sqrt{\frac{1}{n}\sum_{i=1}^{n}{\left({\mathrm{PVC}}_{\mathrm{Method}1,i}-{\mathrm{PVC}}_{\mathrm{Method}2,i}\right)}^{2}}$$

in which *n* is the number of measured PVC values in the pooled A and B scans. These “distances” were plotted in a colour coded distance matrix. Finally, we performed similar consistency checks as for the training dataset.

## 3 Results

### 3.1 Training dataset

**Fig. 1:**
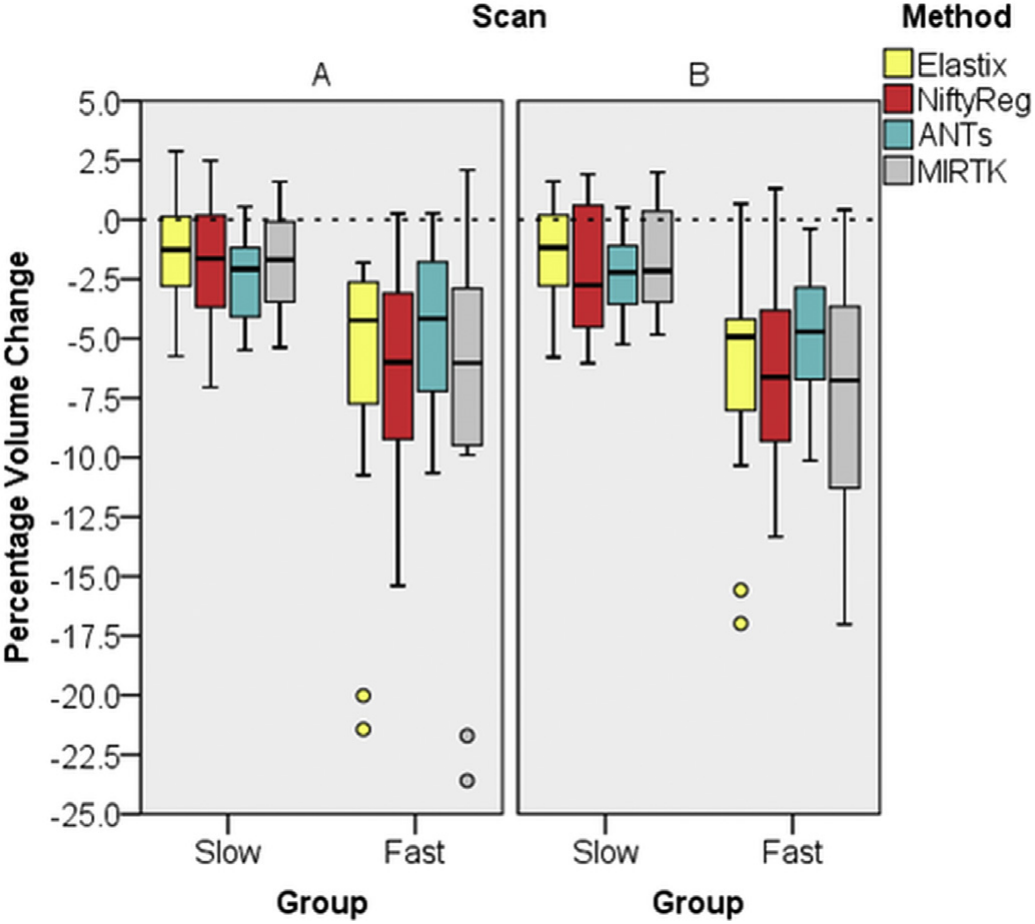
Two-year hippocampal PVC determined with four registration methods for the A and B longitudinal scans. FSL-FIRST BL segmentation was used to determine PVC with the registration methods. Both groups are subjects with MCI, one group with ‘slow’ and one with ‘fast’ progressing atrophy. The small circle are outliers defined by the SPSS software.

Optimized parameter settings can be found in the supplementary files. For all methods, the PVC measured was below our set threshold of ±25%. PVC results obtained with Elastix, NiftyReg, ANTs or MIRTK of the training data set are shown in Fig. 1, where results have been separated in ‘slow’ and ‘fast’ groups. Standard deviations of PVC for pooled A and B measured hippocampal atrophy are presented in Table 1. Mean and SD PVC for the A and B scans separately can be found in the supplementary table 1. Despite the low sample size (N = 16 for each box), median and interquartile ranges overlap well for all registration methods. The 5% volume loss in two years found by all methods is in agreement with the annual hippocampal atrophy of approximately 2.5% found in a meta-analysis by [Tabatabaei-Jafari et al., 2015]. All methods showed a similar PVC trend in the ‘slow’ and ‘fast’ group as determined by repeated measures ANOVA (Table 1). ANTs showed the smallest group differences, while also having the lowest SD.

**Table 1:** Mean (μ) and standard deviation (σ) of two-year hippocampal PVC determined with four registration methods for pooled A and B longitudinal scans. Both groups are subjects with MCI, one group with ‘slow’ and one with ‘fast’ progressing atrophy. Using repeated measures ANOVA significance between the ‘slow’ and the ‘fast’ group was determined. The F-statistic is the ratio of the between group variance and the within group variance and the numbers in the brackets are the degrees of freedom.

Method	Slow		Fast			
	μ	σ	μ	σ	F(1,30)	p-value
Elastix	−1.36	2.153	−6.33	5.448	11.82	0.002
NiftyReg	−1.96	2.781	−6.24	4.154	12.91	0.001
ANTs	−2.39	1.721	−4.66	3.050	7.04	0.013
MIRTK	−1.75	2.218	−7.32	6.124	12.26	0.001

**Fig. 2:**
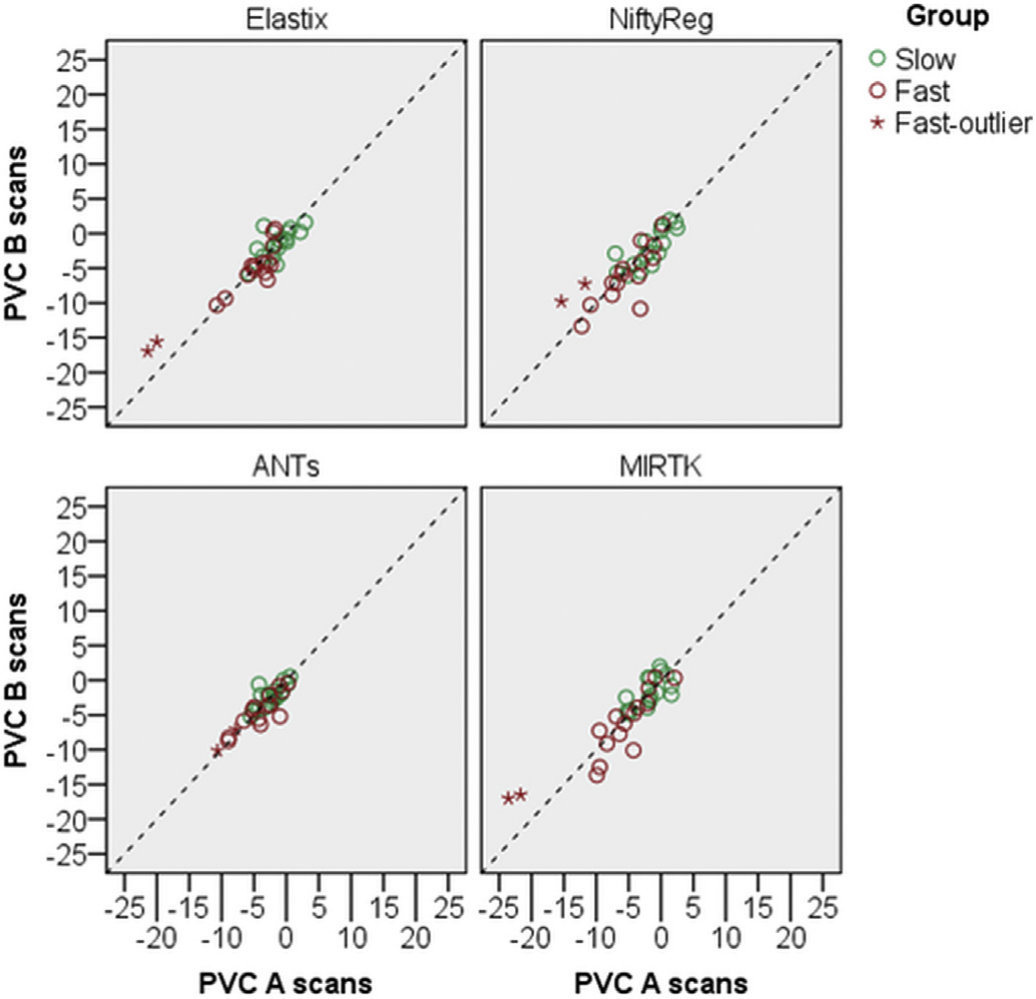
For all four registration methods, hippocampal PVC measured with the A scans is plotted against hippocampal PVC measured with the B scans. The dashed line is the identity line. For Elastix and MIRTK there was one subject in the ‘fast’ group with larger PVC compared to the other cases (two stars correspond to left and right hippocampal PVC). This subject was specifically highlighted (Fast-outlier) to observe if the other methods measured similar high hippocampal atrophy.

In Fig. 2 we plotted hippocampal PVC measured with the A scans against hippocampal PVC measured with the B scans for the training dataset. The average distance (D_Ave_) between A and B scans measured atrophy rates is shown in Table 2. All non-linear registration methods show a similar reproducibility trend with D_Ave_ ranging from 0.86% to 3.08%. ANTs reproduced data best with D_Ave_ = 0.86%.

**Table 2:** Calculated average distance defined in (2) by using mean and standard deviation of pooled (‘slow’ and ‘fast’) hippocampal PVC. All units are %.

A Scans			B Scans		
Method	μ	σ	μ	σ	D_Ave_
Elastix	−3.88	5.269	−3.81	4.391	1.93
NiftyReg	−3.98	4.373	−4.22	3.913	2.88
ANTs	−3.48	2.832	−3.57	2.629	0.86
MIRTK	−4.52	5.780	−4.55	5.005	3.08

#### 3.1.1 Consistency checks with the training dataset

For all ANTs deformation fields, we computed the Jacobian determinants and determined hippocampal PVC by integrating local Jacobian determinants within the region of FSL-FIRST BL hippocampus segmentations. These PVC values were plotted against the PVC values obtained by deforming FSL-FIRST hippocampus meshes (supplementary fig. 1). PVC measured with Jacobian integration was nearly identical to PVC measured by deforming the meshes (linearly fitted line equation: y = −0.03 + 1.00*x and a D_Ave_ of 0.0027%), illustrating the consistency of the mesh and volume-based methods. BL hippocampus segmentations were also computed with MALF and converted to meshes. Using the ANTs deformation fields, we deformed MALFs' BL hippocampal meshes and plotted these hippocampal PVCs against PVCs obtained by deforming FSL-FIRST BL hippocampal meshes (supplementary fig. 2). Both determinations were highly correlated, with a R^2^ obtained from a linearly fitted line of 0.915 and a D_Ave_ of 0.31%. To compare FSL-FIRST and MALF BL segmentations were, we calculated the Jaccard index (Jacc = (A∪B)/(A∩B)) between both BL segmentations. The mean Jaccard indices for the ‘slow’ and the ‘fast’ groups were Jacc_slow_ = 0.70(±0.056) and Jacc_fast_ = 0.73(±0.029) respectively.

#### 3.1.2 Validation dataset

For the validation dataset, FSL-FIRST's segmentation failed in 35 subjects out of 80, which was surprising because in the training dataset FSL-FIRST did not fail. To include these cases, we used FSL's brain extraction tool (BET, [Popescu et al., 2012]) to extract the brains of all subjects and ran FSL-FIRST again. This second FSL-FIRST run still failed in six subjects. These cases were different from the first FSL-FIRST run. To address this incongruity, we included the segmentation files of these 6 subjects from the first FSL-FIRST run for our analysis. In nine subjects, FSL-FIRST exceeded our set PVC threshold of ±25% in which four subjects were from the A scans (3 CTRL, 1 AD) and five from the B scans (2 CTRL, 1 MCI, 2 AD). For one subjects' A and B scans, MALF and Elastix also exceeded the ±25% threshold.

**Fig. 3:**
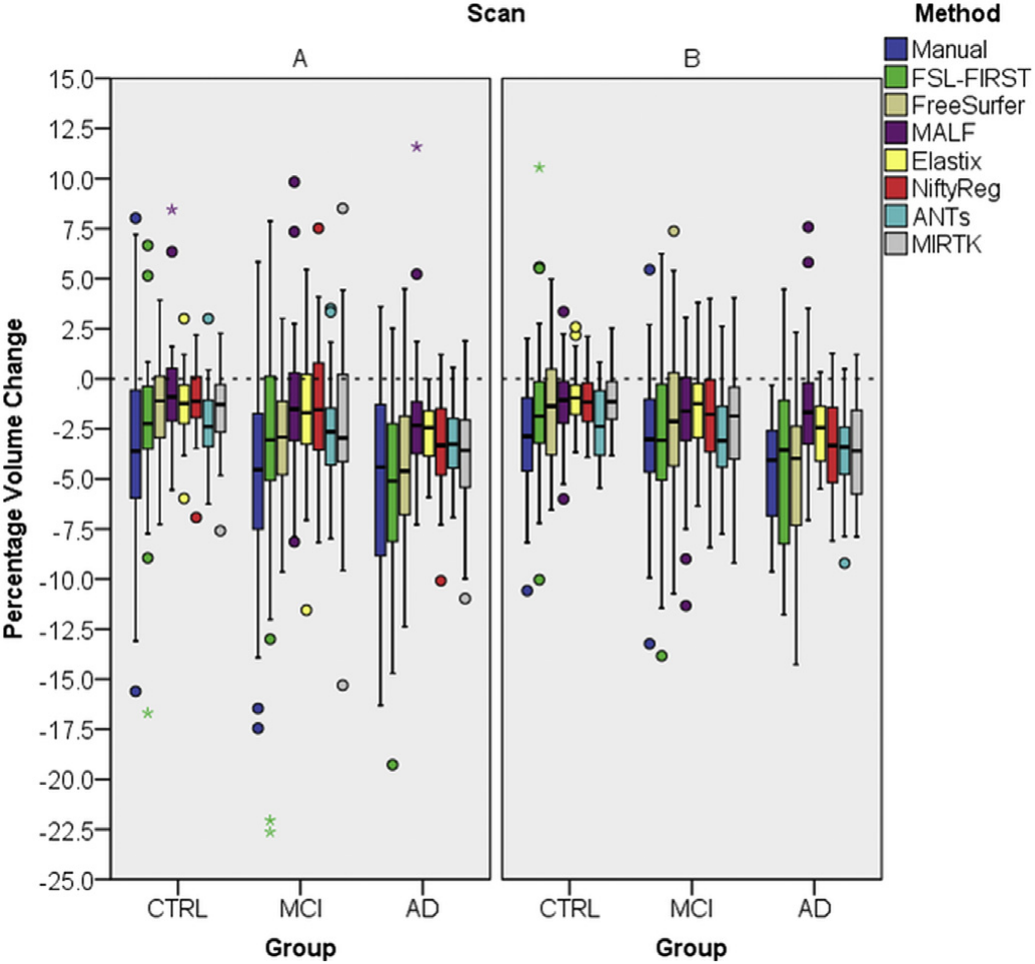
One-year hippocampal PVC measured with eight methods for the A and B longitudinal scans. PVC was determined separately for the CTRL, MCI and AD groups. The small circle and the star sign are outliers and far outliers defined by the SPSS software.

Results of PVCs computations are presented in Fig. 3, separated for the three diagnostic groups and for A and B scans. Corresponding mean and SDs for pooled A and B scans' PVC are given in Table 3 and mean PVC for A and B scans separated can be found in the supplementary table 3. Table 3 also includes results of the repeated measures ANOVA and post hoc analysis, in which PVCs are compared between diagnostic groups for each method. Manual, FSL-FIRST and FreeSurfer showed larger PVC variability compared to the other methods, whereas FSL-FIRST had largest standard deviations (Table 3). Except for MALF, all other methods showed a statistically significant difference between groups using the repeated measures ANOVA. The post hoc analysis for MALF was therefore irrelevant, but for completeness results are presented in Table 3. Tukey post hoc analysis revealed that for all methods there was no significant difference between the CTRL and the MCI groups. All methods except MALF showed statistically significant higher atrophy rates in the AD group compared to the CTRL group.

**Table 3:** For all methods mean (μ), standard deviations (σ) of one-year PVC measured on pooled A and B scans are shown. Using repeated measures ANOVA overall group significance was determined and with Tukey's post-hoc analysis in between group differences were analysed. The F-statistic is the ratio of the between group variance and the within group variance and the numbers in the brackets are the degrees of freedom.

							Overall group significance		post-hoc analysis	
Method	μ_CTRL_	σ_CTRL_	μ_MCI_	σ_MCI_	μ_AD_	σ_AD_	F(2,157)	p-value	p_CTRL-MCI_	p_CTRL-AD_
Manual	−3.18	3.838	−3.89	3.813	−4.81	3.833	3.15	0.045	0.416	0.036
FSL-FIRST	−1.88	3.785	−3.00	4.592	−4.94	4.506	5.59	0.003	0.681	0.005
FreeSurfer	−1.42	2.650	−2.53	3.335	−4.47	3.598	10.71	0.000044	0.137	0.000032
MALF	−0.86	2.121	−1.60	2.737	−1.88	3.145	2.04	0.134	0.243	0.138
Elastix	−1.08	1.480	−1.60	2.359	−2.70	1.665	7.941	0.001	0.262	0.0004
NiftyReg	−1.06	1.516	−1.68	2.719	−3.25	2.474	11.02	0.000033	0.301	0.00004
ANTs	−2.27	1.798	−2.90	2.216	−3.49	1.941	4.01	0.02	0.215	0.014
MIRTK	−1.30	1.752	−2.32	3.133	−3.90	2.599	12.49	0.000009	0.068	0.000006

For FSL-FIRST, Elastix and MALF outliers were removed for the analysis resulting in F_FIRST_(2145), F_Elastix_(2155), F_MALF_(2155)

Results from our sample size calculation defined in eq. (3) are presented in Table 4. Because we grouped A and B scans and left and right hippocampi together, means and the pooled standard deviations for the sample size calculation were obtained from 80 CTRL, 160 MCI and 80 AD samples (for FSL-FIRST, Elastix and MALF some outliers were discarded). Between the CTRL and MCI group FreeSurfer's and MIRTK's clearly had lowest estimated sample sizes (N_CTRL-MCI_) with approximately 30% to 40% less samples required than ANTs, N_CTRL-MCI_ = 162. For N_CTRL-AD_ FreeSurfer, Elastix, NiftyReg and MIRTK showed lowest estimated sample sizes with approximately 55% to 70% less samples required than ANTs estimated sample sizes (N_CTRL-AD_ = 37). This is about 20% of the number of samples used in this study.

**Table 4:** Estimated Cohen's d effect size and minimal sample size required to detect difference between groups for all methods as defined in Eq. (3). Lowest sample sizes are highlighted with bold font.

	Cohen's d effect size		Sample size	
Method	d_CTRL-MCI_	d_CTRL-ADI_	N_CTRL-MCI_	N_CTRL-AD_
Manual	0.19	0.42	452	87
FSL-FIRST	0.27	0.74	222	29
FreeSurfer	0.37	0.96	**115**	**17**
MALF	0.30	0.38	174	108
Elastix	0.26	1.03	226	**15**
NiftyReg	0.29	1.07	193	**14**
ANTs	0.31	0.65	162	37
MIRTK	0.40	1.17	**97**	**11**

**Fig. 4:**
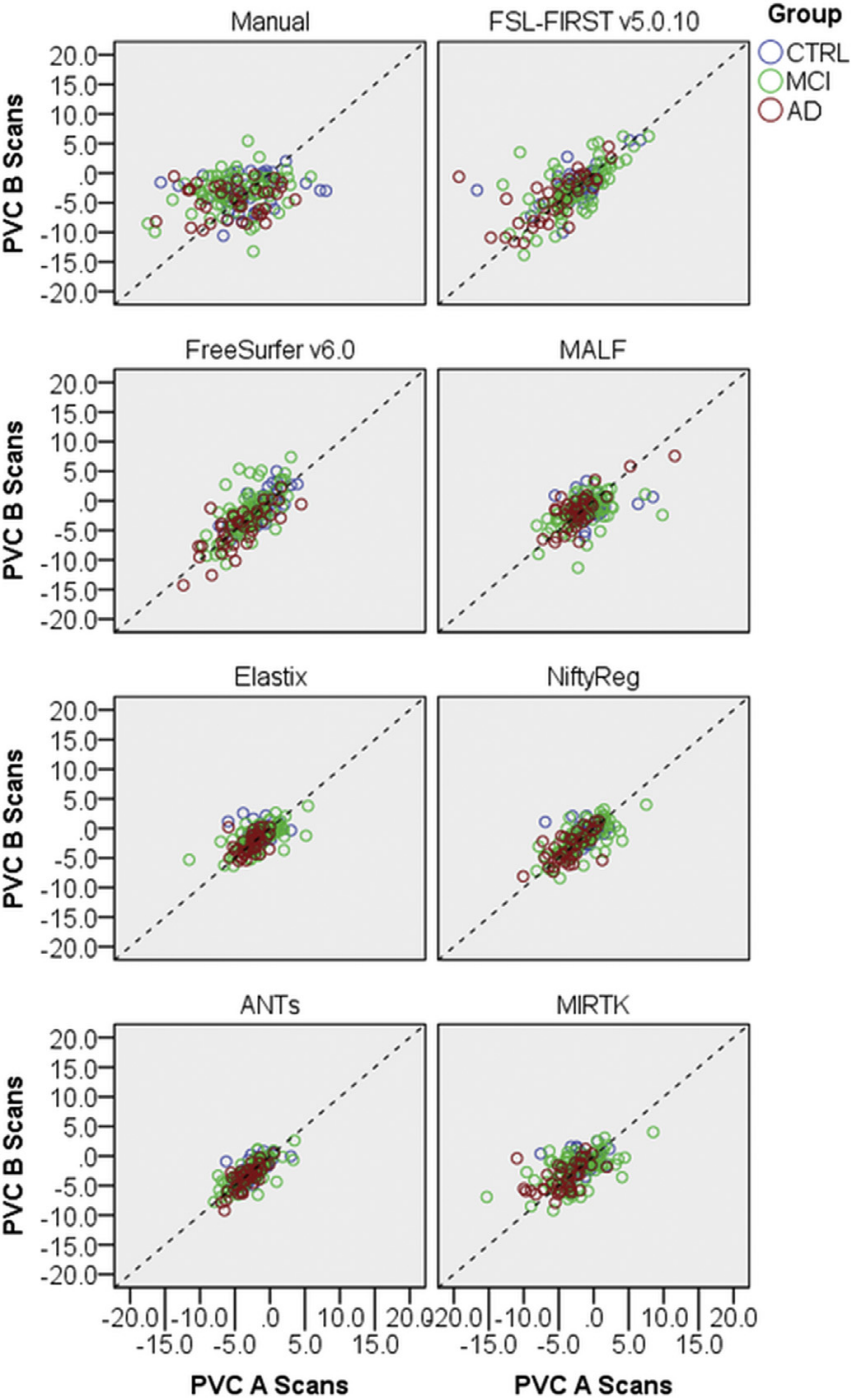
For all eight methods, one-year hippocampal PVC determined with the longitudinal A scans is plotted against hippocampal PVC determined with the longitudinal B scans. The dashed line is the identity line.

In Fig. 4, PVC determined using the A scans was plotted against those based on the B scans. Note that the figure looks ‘noisier’ than Fig. 2 because here one-year atrophy rates are used, while the two-years rate is presented in Fig. 2. Table 5 presents corresponding D_Ave_. ANTs had lowest D_Ave_ (1.06%) followed by Elastix (1.78%) and NiftyReg (2.11%). Manually determined atrophy rates showed poorest reproducibility, D_Ave_ = 12.39%. Using the same hippocampus segmentations and MRI scans but a different statistical analysis, Mulder and colleagues observed similarly poor reproducibility based on manual segmentations [Mulder et al., 2014].

**Table 5:** Calculated average distance defined in (2) by using mean and standard deviation of pooled (CTRL, MCI and AD) hippocampal PVC. All units are %.

	A Scans		B Scans		
Method	μ	σ	μ	σ	D_Ave_
Manual	−4.40	4.574	−3.49	2.914	12.39
FSL-FIRST	−3.33	4.398	−2.93	3.962	6.15
FreeSurfer	−2.92	3.150	−2.56	3.672	3.26
MALF	−1.34	2.908	−1.62	2.524	3.60
Elastix	−1.79	2.243	−1.64	1.898	**1.78**
NiftyReg	−1.89	2.647	−1.95	2.428	**2.11**
ANTs	−2.84	2.097	−2.93	2.088	**1.06**
MIRTK	−2.62	3.163	−2.30	2.525	3.76

**Fig. 5:**
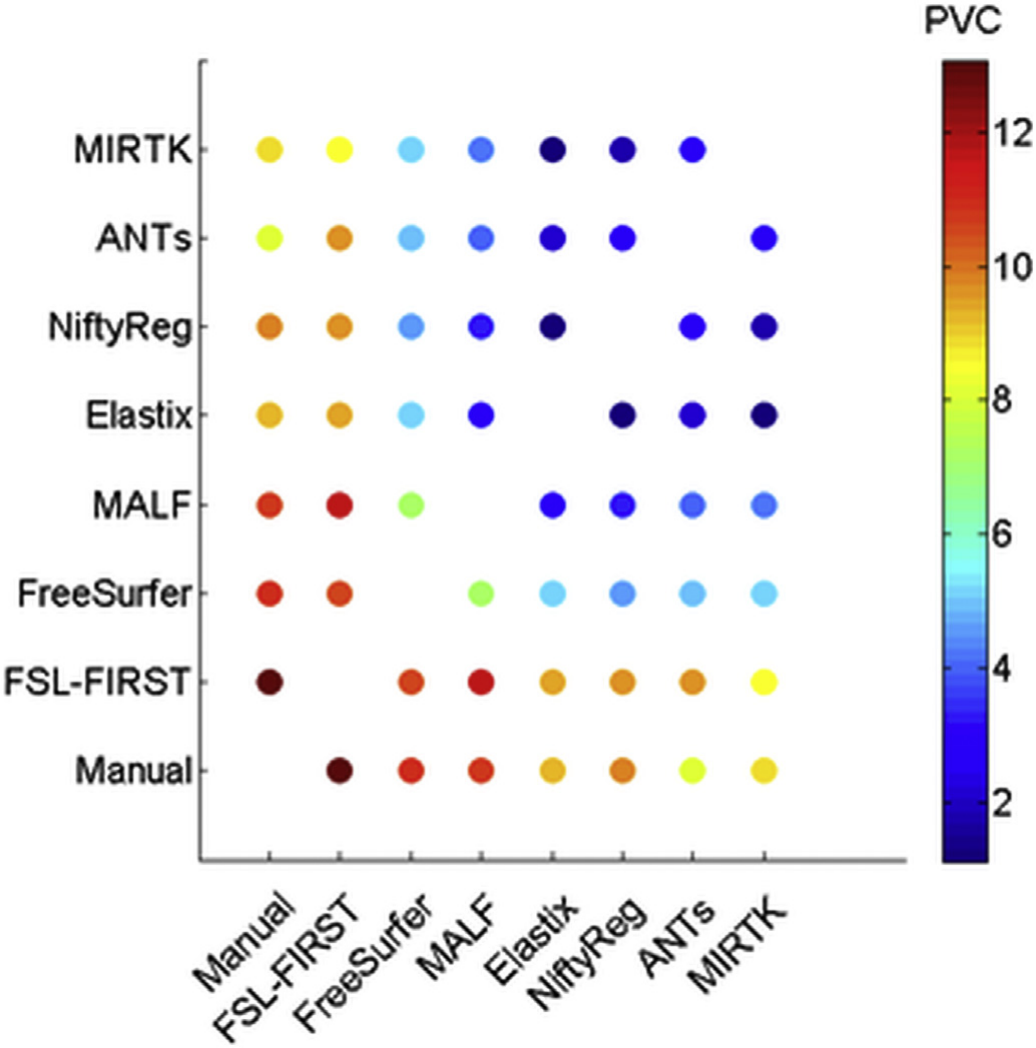
Using the one-year hippocampal PVC of the validation dataset, the average “distance” of atrophy rate agreement was calculated using (2) and plotted in a colour coded matrix. Atrophy measurement methods with lower distance in PVC (shown in %) have a better correspondence.

The distance correlation matrix used to visualize overall differences between methods is shown in Fig. 5 and the corresponding numerical average distances in supplementary table 3. These distances show that atrophy determined manually or with FSL-FIRST correlated poorly with other methods.

#### 3.1.3 Consistency checks with the validation dataset

PVC values for the non-linear registration methods were determined by deforming manual BL segmentations (PVC_Manual_ANTs_). To assess dependency on BL segmentation we also used BL segmentations from MALF and deformed these with ANTs deformation fields to obtain new PVC values (PVC_MALF_ANTs_). PVC_Manual_ANTs_ was plotted against PVC_MALF_ANTs_ (supplementary fig. 3). The R^2^ was 0.837 and the average distance was 0.46%. For the outlier AD cases (red circles in supplementary fig. 3), which are further away from the identity line, we inspected the BL segmentations. MALF BL segmentations slightly overestimated the hippocampal region and also outlined a small part of the cerebral fluid next to the hippocampal boundaries. Removing these cases yielded a R^2^ of 0.927 and a D_Ave_ of 0.26%, illustrating that BL segmentations are largely interchangeable for computing atrophy rates.

## 4 Discussion

In this study we compared hippocampal atrophy rates determined using eight different methods and evaluated their differences based on plausibility over diagnostic groups, their estimated errors and their reproducibility over back to back scans. All methods showed largest PVCs for AD, followed by MCI and controls. The non-linear registration-based FreeSurfer and MIRTK showed highest sensitivity in terms of predicted sample size, and ANTs, Elastix and NiftyReg showed largest reproducibility. The segmentation-based technique FSL-FIRST and manual segmentations scored lowest in these aspects.

Our atrophy rates reproducibility analysis is in agreement with [van de Pol et al., 2007a] who used directional non-linear registration (biased) to measure hippocampal atrophy rates and showed that hippocampal atrophy measured with non-linear registration is more reproducible than manually measured atrophy. We investigated this with multiple symmetric non-linear registration (unbiased) methods and automatic segmentation methods, and we also conclude that hippocampal atrophy rates should preferably be computed using such methods. Using manual segmentation, large sample sizes are needed to detect differences between diagnostic groups.

For the validation dataset, FSL-FIRST had several failed segmentations and MALF was not able to detect hippocampal atrophy rate differences between diagnostic groups. For FSL-FIRST, some of the failed segmentations were caused by poor internal registration results indicating that the procedures might be susceptible to noise. MALF's poorer performance might be caused by the label fusion, averaging out the subtle differences between segmentations.

The ANOVA analysis (Table 3) showed that none of the methods could find a significant difference in hippocampal atrophy between the CTRL and MCI groups, while the closest was method was MIRTK (p-value = .068). One possible reason for this could be that we merged two MCI subgroups together, as described in section 2.1 Validation dataset. Only selecting MCI subjects with AD-positive cut-off values (tau/Aβ_1–42_ ≥ 0.39) might result to a clearer differentiation between the CTRL and MCI groups. Between CTRLs and AD a significant group difference was detected by all methods (MALF excluded).

Except for ANTs, for N_CTLR-AD_ non-linear registration-based methods resulted in lowest estimated samples sizes. ANTs determined on average almost 1% more hippocampal atrophy in the CTRL and MCI group (Table 3), which explains the higher required sample size of N_CTLR-AD_ = 37. Only looking at the sample size analysis would suggest using MIRTK or FreeSurfer. We performed the reproducibility analysis to get a better understanding of the performance and reliability of the methods. This analysis showed that from the non-linear registration-based methods the reproducibility rates of MIRTK in the validation dataset were the worst (D_Ave_ = 3.76%) and ANTs reproducibility rates the best (D_Ave_ = 1.06%). Reproducibility of hippocampal atrophy rates is an important quality measure and because the differences between best performing methods are small, our results suggest using either ANTs, Elastix or NiftyReg to determine hippocampal atrophy rates. Since the differences between those methods are fairly small, the preferred method would mostly depend on computational efficiency.

The conclusions above are also reflected in the colour coded distance matrix, where one can observe that the registration-based methods, including MALF and FreeSurfer, agree best with each other. Within those, Elastix, MIRTK and NiftyReg had smallest average distances ranging from 1.1% to 1.7%, possibly reflecting that these are based on the same underlying principle (FFD). Comparing ANTs to these methods yielded average distances ranging from 2.2% to 2.9%.

In the validation dataset we deformed manual BL segmentations with ANTs to determine hippocampal atrophy rates. But we also showed that when replacing manual BL segmentation by MALF or FSL-FIRST segmentation in the validation dataset, atrophy rates were hardly affected (supplementary fig. 2 and 3), showing that the accuracy of the BL segmentations is not vital for the outcome of non-linear methods. Therefore, methods to measure hippocampal atrophy rates that are based non-linear registration methods can be completely automated by supplying an automatic BL segmentation. Such a procedure is similar to FreeSurfer's longitudinal pipeline, in which images are first pre-processed (resampling, skull stripping, intensity normalization), then an unbiased atlas registration procedure is used and finally labels from different time-points are fused (Reuter et al., 2012, 2010; Reuter and Fischl, 2011).

Of all automatic segmentation methods (FSL-FIRST, MALF and FreeSurfer), FreeSurfer performed best. We used FreeSurfer v6.0 and compared to other studies in which v5.3 was used [Cover et al., 2016; Mulder et al., 2014], FreeSurfer clearly improved and atrophy rates were well reproduced in comparison with the other methods (4th lowest of all methods; D_Ave_ = 3.26%). It is important to note again that different software may select different parts of the hippocampus, and some may even in some cases include other tissues. It will be important for future studies to investigate this heterogeneity and establish harmonized protocols, such as HarP (http://www.hippocampal-protocol.net/), for the assessment and reparation of errors.

Somewhat surprisingly given the previous good performance of Multiple-Atlas Propagation and Segmentation with Hippocampal Boundary Shift Integral (MAPS-HBSI) in [Cover et al., 2016] is the poor performance of the multi-atlas method MALF. This discrepancy could be due to the fact that the registration used in our implementation of MALF was fast but not maximally optimized, and to the fact that MAPS-HBSI included a boundary shift integral calculation to compute volume change, whereas in this paper only the BL and FU cross-sectional segmentations were used to calculate volume change. Furthermore, different label fusion techniques might improve results for multi-atlas segmentation methods as for example in [Song et al., 2017; Zhu et al., 2017].

Manual segmentation was performed on resliced MRI with 2 mm slices. Supplementary fig. 3 and the corresponding analysis illustrated that this reslicing did not have a large influence in our analysis, because MALF was performed on native MR image resolution. The low reproducibility of manually measured atrophy rates was to some extent surprising, because hippocampus segmentations of the A and B scans showed high outline reproducibility (Jaccard~0.8 equivalent to a Dice~0.89 and ICC for absolute agreement of 0.98) as found in a previous study using the same manual segmentations [Bartel et al., 2017]. Apparently, such small differences in outlines with uncorrelated errors result in large uncertainties in volume. The reason why non-linear registration-based methods are more accurate is that registrations work at sub-pixel level, whereas manual segmentations are outlined pixel-by-pixel and contain therefore more noise. This noise is enhanced in the relative difference, making manual segmentation a poor choice for measuring atrophy rates.

Even in elderly healthy aging subjects, hippocampal atrophy is to be expected [Fraser et al., 2015]. However, in all groups (CTRL, MCI and AD) and for all methods we also measured unexpected hippocampal volume increase for a few subjects (Fig. 3). Unexpected hippocampal volume increase for a few subjects can also be observed in other studies in which hippocampal atrophy rates were measured [Cover et al., 2016; Mulder et al., 2014; van de Pol et al., 2007a; Yushkevich et al., 2010]. We did not further investigate these subjects with positive PVCs, but most probably they are due to noise in the images.

Each registration method might be improved by adjusting registration parameters. We used the training dataset and changed parameters until we measured similar group-based atrophy rates for each method. A larger investigation to obtain optimal registration parameters would be preferable but is also challenging because a ground truth does not exist, since manual segmentations are not reliable enough.

Except of brain extraction to reduce image registration time, we did not include any other image pre-processing steps. Intensity normalization might improve results for all non-linear registration methods. In FreeSurfer's pipeline intensity normalization is already included. Further improvements may be achieved by removing directional registration bias during the global/rigid registration step and by applying a symmetric global/rigid registration as discussed and investigated in [Modat et al., 2014; Yushkevich et al., 2010].

### 4.1 Clinical applicability

Our findings are of interest for clinical trials enrolling MCI and AD subjects to aim for a treatment in earlier parts of the disease process. In such trials hippocampal volume change plays a role in the inclusion and exclusion criteria and is an important secondary outcome measure (Albert et al., 2011; Dubois et al., 2014; Hill et al., 2014). We determined consistent methods, for which low sample sizes of approximately 15–35 subjects are needed to determine a significant difference between CTRL and AD subjects. Because the volume change difference between CTRL and MCI subjects is smaller, many more subjects are needed for such trials. However, compared to manually determined hippocampal volume change, for the most consistent methods, such as ANTs, Elastix and NiftyReg, 64%, 50% and 57% less samples are needed respectively.

Furthermore, we believe that our findings have potential clinical applicability in radiotherapy, where hippocampal volume loss has recently become a point of concern when patients are given prophylactic cranial irradiation (PCI). In a recent study, longitudinal brain changes were reported in 22 patients with SCLC received PCI using the SPM software package (http://www.fil.ion.ucl.ac.uk/spm/) [Simó et al., 2016]. There, hippocampal volume decrease was associated with PCI, but the magnitude of hippocampal volume loss was not reported. In a small case series study (n = 9) patients with melanoma brain metastasis showed a mean hippocampal volume loss of 7.81% due to whole brain irradiation therapy after 6 month [Hong et al., 2017]. Additionally, in patients with primary brain tumours dose was significantly correlated with hippocampal volume loss one year after radiotherapy, while high doses of >40Gy showed a mean hippocampal volume loss of 5.8% and low doses of <10Gy a mean volume loss of 1.2% [Seibert et al., 2017]. These reported hippocampal volume losses exceed one-year hippocampal atrophy in AD of 2.5–3.5% as determined in this study. Hence, we expect that radiation induced annual hippocampal volume loss should be detectable with for instance ANTs, Elastix or NiftyReg even with fairly small sample size (Table 4).

## 5 Conclusion

Hippocampal volume loss measured on longitudinal T1-weighted MRI should preferably be computed with symmetric non-linear registration methods such as ANTs, Elastix or NiftyReg, because these were least susceptible to noise. Such methods allow detecting the difference in one-year atrophy between Alzheimer patients and healthy controls with approximately 15–35 patients.

## Appendix A Supplementary data

Supplementary material Image 1
