# Supplementary material for: Non-linear registration improves statistical power to detect hippocampal atrophy in aging and dementia
Source: Neuroimage Clin. 2019 Jun 18;23:101902. doi: 10.1016/j.nicl.2019.101902 (PMC6595082; doi:10.1016/j.nicl.2019.101902)
Supplement: Supplementary file 1 — Supplementary material [file mmc1.docx]

# Supplementary Files

**Supplementary Tables:**

Supplementary Table 1: Mean (μ) and standard deviation (σ) of two-year hippocampal PVC determined with four registration methods for the A and B longitudinal scans. Both groups are subjects with MCI, one group with ‘slow’ and one with ‘fast’ progressing atrophy.

| Scan | Method | Slow | | Fast | |
| --- | --- | --- | --- | --- | --- |
|  |  | μ | σ | μ | σ |
| A | Elastix | -1.27 | 2.311 | -6.49 | 6.125 |
|  | NiftyReg | -1.76 | 2.934 | -6.20 | 4.520 |
|  | ANTs | -2.47 | 1.835 | -4.49 | 3.322 |
|  | MIRTK | -1.76 | 2.324 | -7.28 | 6.886 |
|  |  |  |  |  |  |
| B | Elastix | -1.46 | 2.054 | -6.17 | 4.874 |
|  | NiftyReg | -2.15 | 2.702 | -6.29 | 3.902 |
|  | ANTs | -2.30 | 1.654 | -4.83 | 2.851 |
|  | MIRTK | -1.74 | 2.183 | -7.37 | 5.486 |

Supplementary Table 2: Mean (μ) and standard deviation (σ) of two-year hippocampal PVC determined with eight methods for the A and B longitudinal scans.

| Scan | Method | μ_CTRL_ | σ_CTRL_ | μ_MCI_ | σ_MCI_ | μ_AD_ | σ_AD_ |
| --- | --- | --- | --- | --- | --- | --- | --- |
| A | Manual | -3.26 | 4.676 | -4.66 | 4.391 | -5.01 | 4.745 |
|  | FSL-FIRST | -2.36 | 3.891 | -3.24 | 5.068 | -5.51 | 4.781 |
|  | FreeSurfer | -1.50 | 2.529 | -2.96 | 2.877 | -4.28 | 3.647 |
|  | MALF | -0.52 | 2.360 | -1.41 | 2.952 | -2.03 | 3.192 |
|  | Elastix | -1.26 | 1.605 | -1.63 | 2.660 | -2.75 | 1.605 |
|  | NiftyReg | -1.09 | 1.587 | -1.59 | 2.893 | -3.30 | 2.495 |
|  | ANTs | -2.30 | 1.817 | -2.87 | 2.324 | -3.31 | 1.777 |
|  | MIRTK | -1.55 | 1.946 | -2.37 | 3.523 | -4.18 | 2.842 |
|  |  |  |  |  |  |  |  |
| B | Manual | -3.10 | 2.822 | -3.13 | 2.964 | -4.60 | 2.675 |
|  | FSL-FIRST | -1.42 | 3.677 | -2.74 | 4.064 | -4.34 | 4.179 |
|  | FreeSurfer | -1.34 | 2.796 | -2.11 | 3.708 | -4.66 | 3.584 |
|  | MALF | -1.20 | 1.821 | -1.78 | 2.509 | -1.73 | 3.134 |
|  | Elastix | -0.87 | 1.338 | -1.57 | 2.031 | -2.65 | 1.742 |
|  | NiftyReg | -1.02 | 1.461 | -1.78 | 2.547 | -3.21 | 2.485 |
|  | ANTs | -2.24 | 1.801 | -2.92 | 2.118 | -3.66 | 2.099 |
|  | MIRTK | -1.05 | 1.518 | -2.27 | 2.710 | -3.62 | 2.332 |

Supplementary Table 3 Average “distance” atrophy rates in % between methods using equation defined in (4).

|  | Manual | FSL-FIRST | FreeSurfer | MALF | Elastix | NiftyReg | ANTs | MIRTK |
| --- | --- | --- | --- | --- | --- | --- | --- | --- |
| Manual | 0.0 | 13.1 | 10.9 | 10.7 | 9.2 | 9.8 | 8.1 | 8.8 |
| FSL-FIRST | 13.1 | 0.0 | 10.5 | 11.6 | 9.3 | 9.6 | 9.6 | 8.6 |
| FreeSurfer | 10.9 | 10.5 | 0.0 | 7.2 | 5.1 | 4.6 | 5.0 | 5.1 |
| MALF | 10.7 | 11.6 | 7.2 | 0.0 | 2.9 | 3.2 | 4.0 | 4.2 |
| Elastix | 9.2 | 9.3 | 5.1 | 2.9 | 0.0 | 1.3 | 2.2 | 1.1 |
| NiftyReg | 9.8 | 9.6 | 4.6 | 3.2 | 1.3 | 0.0 | 2.6 | 1.7 |
| ANTs | 8.1 | 9.6 | 5.0 | 4.0 | 2.2 | 2.6 | 0.0 | 2.9 |
| MIRTK | 8.8 | 8.6 | 5.1 | 4.2 | 1.1 | 1.7 | 2.9 | 0.0 |

**Supplementary Figures:**


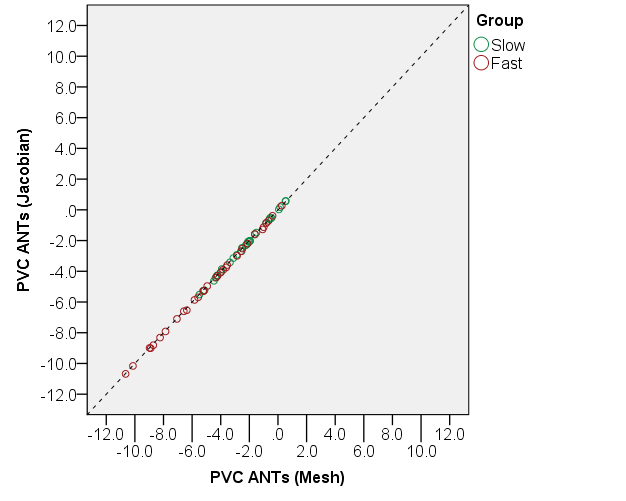


Supplementary Figure 1: For the training dataset FSL-FIRST baseline (BL) hippocampus segmentations were used to measure two-year PVC in two ways: 1) The deformation field obtained from ANTs non-linear registration was applied on BL hippocampal meshes and percentage volume change (PVC) was calculated between the BL and the deformed mesh. 2) Local Jacobian determinants of the deformation field was integrated in the BL hippocampus segmentation area to measure PVC. Both PVC measurements were plotted against each other. The dashed line is the identity line.


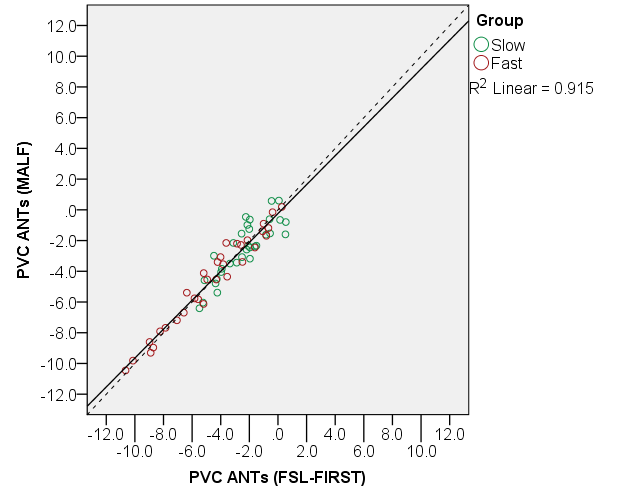


Supplementary Figure 2: For the training dataset FSL-FIRST and MALF baseline (BL) hippocampus segmentations were available for the same subjects. These segmentations were converted to meshes and mapped to follow up image using deformation fields obtained from the ANTs registrations. Percentage volume change (PVC) was calculated and ANTs PVC using FSL-FIRST BL segmentation was plotted against ANTs PVC using MALF BL segmentation. The dashed line is the identity line and the solid line a linearly fitted line.


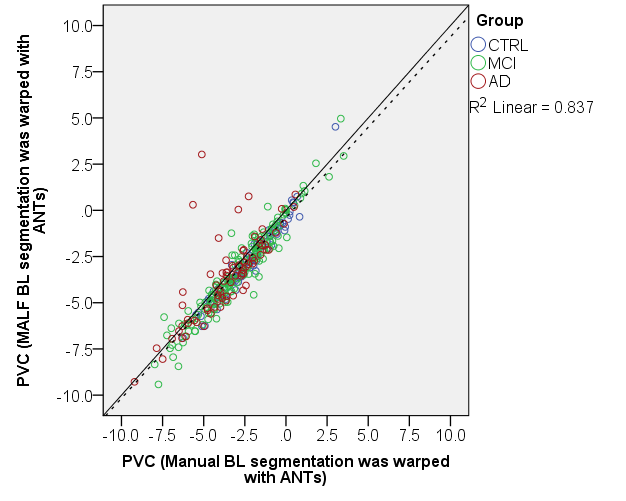


Supplementary Figure 3: Manual baseline (BL) and MALF BL segmentations were warped to follow up images and percentage volume change (PVC) was calculated and plotted against each other. In this figure one subject was removed due to a failed segmentation (MALF, described in the result section). The solid line is the identity line and the dashed line is the linearly fitted line. The average distance was 0.46%. For the outlier AD cases (red circles) which are further away from the identity line, we inspected the BL segmentations. MALF BL segmentations slightly overestimated the hippocampal region and also outlined a small part of the cerebral fluid next to the hippocampal boundaries. Removing these yielded a R^2^ of 0.927 and a D_Ave_ of 0.26%.

*Parameter settings for MALF:*

antsJointLabelFusion.sh -d 3 -c 2 -j 8 -q 0 -x or -t $target -o $subj_base -p ${subj_base}_posteriors%02d.nii.gz –g $atlas1 –l $label1 –g $atlas2 –l $label2 …

antsJointLabelFusion.sh used antsRegistrationSyn.sh when the –q flag is 1, otherwise the antsRegistrationQuickSyn.sh is used. antsRegistrationQuickSyn.sh uses the same registration scheme as antsRegistrationSyn.sh, but the metric used in antsRegistrationQuickSyn.sh is mutual information (MI) with 32bins. This makes the registration much fast than antsRegistrationSyn.sh, which uses cross-correlation (CC) with a radius of 4.

*Parameter settings for Elastix v4.801:*

For Elastix’s groupwise registration (symmetric registration) images need to be merged into 4D volumes. The underlying principle is described in detail [Metz et al., 2011]. First we resampled the source image to target image using FSL-FLIRT rigid registration and spline interpolation. These images were then merged to a 4D MRI image (4DMRI). The brain mask from the target and source image were combined, duplicated and the two duplicated images were also merged to a 4D MRI image (4DBrainMask). Using these 4D images, calling Elastix with a specific parameter file transforms both images of the 4DMRI to a mean space. To obtain the transformation from time-point 0 to time-point 1, the inverse transformation also need to be computed. Elastix transform parameters file was adapted from [Metz et al., 2011], in which we specifically needed to lower the control point spacing (FinalGridSpacingInPhysicalUnits 4).

“Forward” registration:

elastix -f ${4DMRI} -m ${4DMRI} -fMask ${4DBrainMask} -p ParameterFile.txt -out ${OutpDir}/

“Inverse” registration:

For inverse transformation the same parameter file was used but the metric was changed to "DisplacementMagnitudePenalty"

elastix -f ${4DMRI} -m ${4DMRI} -t0 ${OutpDir}/TransformParameters.0.txt -p $ParameterFile_Inv.txt -out ${OutpDir}/

*Parameter settings for NiftyReg v1.4.0:*

For NiftyReg we used the –vel flag for symmetric registration and varied the control point spacing ending up with 2.5mm in all dimensions. All other parameters were default: -be 0.005, -le 0.0 0.0 –l2 0.0 –jl 0.0 –maxit 300 –ln 3 –nmi 64bins.

reg_f3d -target target.nii -source source.nii –rmask target_mask.nii –fmask source_mask.nii –aff initial_registration_parameters.txt –cpp source-to-target_cpp.nii –vel –sx 2.5

*Parameter settings for ANTs v2.2.0:*

For ANTs we used similar registration parameters as used in ANTs cortical thickness measurement script (https://github.com/ANTsX/ANTs/blob/master/Scripts/antsCorticalThickness.sh), but multi-resolution features were not used and the number of iterations was set to 60 adapted from [Yushkevich et al., 2010] and [Das et al., 2012]. This lead to the following command line:

antsRegistration -d 3 -r $initial_registration_parameters.tfm -m CC[${target},${source},1,4] -t SyN[0.1,3,0] -c [60,1.e-6,10] -f 1 -s 0 -l 1 -z 1 -x [${target_brain_mask}, ${source_brain_mask}] -o [${basename},${basename}_imgwarp.nii]

*Parameter settings for MIRTK (*compiled from the git development tree https://github.com/schuhschuh/MIRTK/tree/develop rev daf2b89, built on Dec 19 2017)*:*

For MIRTK we varied the control point spacing and the bending energy weight.

mirkt register -parin $parameter_file -image $target -image $source -dof_i ${initial_registration_parameters.dof -dofin identity -dofout ${basename}_mirtk-svffd.dof.gz

Parameter file for Elastix:

// *********************

// * ImageTypes

// *********************

(FixedInternalImagePixelType "short")

(MovingInternalImagePixelType "short")

(FixedImageDimension 4)

(MovingImageDimension 4)

(UseDirectionCosines "true")

// *********************

// * Components

// *********************

(Registration "MultiResolutionRegistration")

(Interpolator "ReducedDimensionBSplineInterpolator")

(ResampleInterpolator "FinalReducedDimensionBSplineInterpolator")

(Resampler "DefaultResampler")

(BSplineInterpolationOrder 1)

(FinalBSplineInterpolationOrder 3)

(FixedImagePyramid "FixedSmoothingImagePyramid")

(MovingImagePyramid "MovingSmoothingImagePyramid")

(Optimizer "AdaptiveStochasticGradientDescent")

(HowToCombineTransforms "Compose")

//Groupwise transform:

(Transform "BSplineStackTransform")

//Groupwise metric:

(Metric "PCAMetric")

//Specific for the LinearGroupwiseMI metric

(TemplateImage "ArithmeticAverage" "ArithmeticAverage")

(Combination "Sum" "Sum")

(UseFastAndLowMemoryVersion "true")

(NumberOfFixedHistogramBins 32)

(NumberOfMovingHistogramBins 32)

//Specific for the PCAMetric

(NumEigenValues 1)

// *********************

// * Groupwise Metric settings

// *********************

(SubtractMean "true")

(MovingImageDerivativeScales 1 1 1 0)

//Choose one of the following settings:

(FinalGridSpacingInPhysicalUnits 4)

// *********************

// * Optimizer settings

// *********************

(NumberOfResolutions 2)

(AutomaticParameterEstimation "true")

(ASGDParameterEstimationMethod "Original")

(MaximumNumberOfIterations 1000)

// *********************

// * Pyramid settings

// *********************

(ImagePyramidSchedule 2 2 2 0 1 1 1 0)

// *********************

// * Sampler parameters

// *********************

(NumberOfSpatialSamples 2048)

(NewSamplesEveryIteration "true")

(ImageSampler "RandomCoordinate")

(CheckNumberOfSamples "true")

// *********************

// * Mask settings

// *********************

(ErodeMask "false")

(ErodeFixedMask "false")

// *********************

// * Output settings

// *********************

(DefaultPixelValue 0)

(WriteResultImage "true")

(ResultImagePixelType "float")

(ResultImageFormat "nii.gz")

Parameter file for MIRTK:

## Input images

Background value = -1

## Transformation model

Multi-level transformation = None

Transformation model = SVFFD

Control point spacing = 2.5

Integration method = SS

No. of integration steps = 32

No. of BCH terms = 2

## Objective function

Energy function = SIM[Dissimilarity](I(1) o T^-0.5, I(2) o T^0.5)...

+ 0 BE[Bending energy](T)...

+ 0 LE[Linear energy](T)...

+ 0 LogJac[LogJac penalty](T)...

+ 0 NegJac[NegJac Penalty](T)

Image dissimilarity foreground = Union

Image dissimilarity measure = NMI

No. of bins = 64

Bending energy weight = 0.05

Bending energy w.r.t. world = Yes

Bending energy use spacing = No

Elastic energy weight = 0

Elastic energy mu = 1

Elastic energy lambda = 0

Elastic energy rotation = Yes

Elastic energy w.r.t. world = Yes

Elastic energy use spacing = No

LogJac penalty weight = 0

LogJac penalty epsilon = 0.01

LogJac penalty domain = Lattice

LogJac penalty w.r.t. world = Yes

LogJac penalty use spacing = No

NegJac penalty weight = 0

NegJac penalty epsilon = 0.1

NegJac penalty threshold = 0.5

NegJac penalty domain = Lattice

NegJac penalty w.r.t. world = Yes

NegJac penalty use spacing = No

## Optimization

Downsample images with padding = Yes

Optimization method = ConjugateGradientDescent

Line search strategy = Adaptive

Divide data terms by initial value = No

Precompute image derivatives = No

Constrain passive DoFs = No

Conjugate total energy gradient = Yes

B-spline FFD gradient calculation = Convolution

No. of resolution levels = 3

Maximum no. of gradient steps = 100

Maximum no. of line search iterations = 12

Maximum streak of rejected steps = 2

No. of last function values = 0

Epsilon = 0
